# Seroprevalence of Infectious Bronchitis Virus in Broiler and Layer Farms of Central Ethiopia

**DOI:** 10.1155/2022/8915400

**Published:** 2022-03-02

**Authors:** Jirata Shiferaw, Tamiru Dego, Misgana Tefera, Yobsan Tamiru

**Affiliations:** ^1^Department of Pathology and Parasitology, College of Veterinary Medicine and Agriculture, Addis Ababa University, Bishoftu, Ethiopia; ^2^Oromia Technical & Vocational Education Training Bureau Holeta Poly Technic College, Ethiopia; ^3^Department of Microbiology, School of Veterinary Medicine, Wollega University, Nekemte, Ethiopia

## Abstract

**Background:**

Infectious bronchitis virus (IBV) is a highly contagious viral disease of chicken typically affecting the reproductive and respiratory tract and results in possible economic causes from its serious infectious and transmission characteristics.

**Methods:**

A cross-sectional study was carried on serum samples of chickens selected from six (two commercial and four small holder) farms and two types of production (broiler and layer) to detect seroprevalence of IBV and its associated risk factors in Bishoftu and Holeta areas of central Ethiopia from June 2021 to September 2021. A total of 354 blood samples were collected and subjected to indirect ELISA test by IBV antibody test kit ((ProFLOK IBV), from ProFLOK Laboratories Inc., (USA)) to detect specific antibodies against IBV.

**Results:**

Overall, 97.46% seroprevalence was identified. From 230 and 124 samples collected from commercial and smallholder poultry farms, 226 (98.26%) and 119 (95.98%) positive results were obtained, respectively. Among the production types of chickens, high seroprevalence (99.31%) was found in layer poultry, and lower seroprevalence (96.17%) was found in the case of broiler chicken. Significant association was observed among different associated risk factors particularly age, sex, breed, and production types of chickens. From the tested chickens, all age groups, species, and farm types have high seroprevalence of IBV. The prevalence of IBV was highly significant (*p* ≤ 0.01) in the study site. The risk factors indicated could have increased infection prevalence, pathogens' economic impact, and disease occurrence.

**Conclusion:**

IBD is complicating factor affecting poultry production systems in the area. Vaccine and biosecurity measures are recommended for the control of IBV. Furtherly, identification and characterization (by using RT-PCR) of persistent serotype of IBV circulating in the field are recommended.

## 1. Introduction

Ethiopia is home for many livestock species and known for domestication of animals since time of civilization. The livestock sector has been contributing considerable portions to the economy of the country and still promising to rally round the economic development of the country. It contributes 12% and 33% of the total and agricultural gross domestic product (GDP), respectively, provides livelihood for 65% of the population, and accounts for 12-15% of the total export earnings. Ethiopia is believed to have the largest livestock population in Africa. An estimate indicates that the country is home for 59.5 million cattle, 30.7 million sheep, 30.2 million goats, and 56.53 million poultry [1]. This group of livestock and poultry involved in light of global challenges such as climate change, population growth, and the urgency of ensuring the availability of nutritious and secure food; the optimization of sustainable livestock production is more important than ever [2].

Based on the number of animals, poultry represents the largest domestic animal and accounts for more than 30% of all animal protein in the world. Poultry represents an important sector in animal production, with backyard flocks representing a large majority, especially in developing countries. In Ethiopia, for example, villagers raise poultry to meet household food demands and as additional sources of income [3]. Ethiopian entrepreneurs are setting up large, intensively managed flocks of exotic breed, particularly in areas close to Addis Ababa [4]. However, this production is mainly based on commercial poultry, which accounts for only 20% of the total poultry population which are in one way or another affected by different types of infectious diseases.

Globally, infectious bronchitis (IB) is an important respiratory viral disease responsible for enormous economic losses to poultry farmers [5]. IB is an acute, highly contagious viral disease of poultry, clinically manifested by respiratory signs like tracheal rales, sneezing, and coughing [6]. The virus also affects the female reproductive tract, causing poor quality of eggs and loss of egg production [7]. Poultry of all ages can be infected by infectious bronchitis virus (IBV) [8]. It is possibly the most economically important viral respiratory disease of chicken after Avian Influenza (AI) and Newcastle disease (NCD). IB is considered as one of the top economically important poultry diseases as it reduces egg production, degrades egg shell quality, and renders less hatchability and poor body weight gain and poor feed conversion ratio (FCR) in broiler [9].

An acute IBV infection can be diagnosed by detection of IBV virus (antigen) itself or the specific antibody response. The most common assays for routine use of virus detection are virus isolation (VI), immunofluorescence assay (IFA), immunoperoxidase assay (IPA), and polymerase chain reaction (PCR) and for antibody detection the haemagglutination inhibition (HI) test, agar gel precipitation test (AGPT), and enzyme-linked immunosorbent assay (ELISA) [10].

The ELISA technique is a sensitive serological method and gives earlier reactions and higher antibody titers than other tests. It is widely used to identify IBV infected flocks (broiler) based on high antibody titers [11]. A number of ELISA tests for IBV antibody detection have been described and used widely [12]. For the reason that it is a simple, rapid, sensitive, and large-scale evaluation tool, ELISA has been used widely in IBV serological profiling [13].

Avian infectious bronchitis is an infectious viral disease that induces huge economic losses. It has a worldwide distribution and a high morbidity rate that may reach 100%. Despite its significant impact on the poultry industry, limited researches were on the status of the disease in Ethiopia [14, 15]. Therefore, the objective of the present study was to determine the seroprevalence of IBV virus in broiler and layer from different poultry farms (Bishoftu and Holeta areas) in central Ethiopia.

## 2. Materials and Methods

### 2.1. Study Area

The study was conducted in two selected (Bishoftu and Holeta) areas of central Ethiopia. Bishoftu is a town located at 47.9 km southeast of Addis Ababa. The site is found at an altitude of 1920 meters above sea level and has a latitude and longitude of 8°45′N 38°59′E. Holeta has a latitude and longitude of 9°3′N 38°30′E and an altitude of 2391 meters above sea level, respectively, and located 22 km west of Addis Ababa [16].

### 2.2. Study Design and Population

The cross-section study was conducted during the period from July 2021 to September 2021 in central Ethiopia at Bishoftu and Holeta towns from two commercial and four smallholder poultry farms. Commercial and small holders were selected randomly based on the facilities found in the farms and presence of layer and broiler production type. Blood samples were collected from randomly selected, apparently healthy chickens (145 from layer and 209 from broiler) with a total of 354 blood samples collected from exotic Bovans and cob chickens breeds. The potential risk factors like information on flock history (age, sex, breed, and production types), history of vaccination, and farming activities in general were recorded before blood sample collection. Accordingly, there was no vaccination history for the chickens against IBV.

### 2.3. Sample Size Determination

The sample size required for this study was determined based on sample size determination in random sampling for an infinite population with an expected prevalence of 64.7% [15] and 5% desired absolute precision.

(1) $$\begin{array}{c} n=\frac{1.96^{2}-\text{Pexp}\left(1-\text{pexp}\right)}{{\text{d}}^{2}}, \end{array}$$

where *n* is the required sample size, Pexp is the expected prevalence, and *d* is the desired absolute precision.

Therefore, based on the above formula, the total sample sizes were calculated to be 354 animals.

However, to check the validity of IBV ELISA result, a validity test was done two times for each sample. In valid IBV ELISA results, the mean optical density (OD) value of positive control serum is greater than 0.250, and the ratio of the mean value of the positive and negative control (ODPC and ODNC) is greater than 3. For the interpretation of the results, a serum sample positive (SP) control ratio was required. If SP value was >0.3, the IBV antibody status was considered positive but ≤0.3 was taken as negative. For each sample, calculate the SP ratio and antibody titer as follows.

#### 2.3.1. Sample Positive (S/P) Ratio

(2) $$\begin{array}{c} \frac{\text{S}/\text{P}=\text{OD sample}-\text{ODNC}}{\text{ODPC}-\text{ODNC}}. \end{array}$$

#### 2.3.2. Antibody Titer

(3) $$\begin{array}{c} \text{Log}10\left(\text{titer}\right)=0.97*\text{Log}10\left(\text{S}/\text{P}\right)+3.449,\\ \text{Titer}=10\text{ Log}10\left(\text{titer}\right). \end{array}$$

### 2.4. Sample Collection and Preservation

Blood samples were collected aseptically from the wing vein of each chicken. About 2-4 ml of blood samples was collected by sterile disposable syringe with 22 gauges 1¼ needle size, and the syringe was kept slanted on racks at an angle of about 45 degrees to facilitate clotting at room temperature overnight and separation of serum. Separated serum was centrifuged at 1500 rpm and transferred into sterile well-labeled cryovial tubes, and the clear serum samples were stored at -20°C refrigerator in the microbiology laboratory of Addis Ababa University College of Veterinary Medicine and Agriculture (AAU-CVMA) until the test was performed. The test was performed in the serology laboratory of National Veterinary Institution (NVI), Bishoftu, Ethiopia.

### 2.5. Laboratory Procedures and Serological Tests

Serological tests were performed by indirect ELISA (enzyme-linked immunosorbent assay) test method to measure antibody titer level against IBV. Test was performed with commercial ELISA kit (ProFLOK IBV), from ProFLOK Laboratories Inc. (USA). All conditions were standardized according to kit manufacturer instructions using precoated ELISA plates and ready to use reagents. Prior to being assayed, at 1 : 500 sample diluent, positive control and negative control were added into antigen-coated microtiter plates, 100 *μ*l of (Goat) Anti-Chicken: HRPO conjugate was added into each well and incubated at room temperature, 23°C for 30 min. After incubation, the plates were washed 5 times with wash buffer, and 100 *μ*l conjugate reagent was added to the antigen-coated plate. The plate was incubated at room temperature 23°C for 30 min. The plates were washed and added and incubated at room temperature 23°C for 15 min after adding 100 *μ*l substrate reagents. Finally, 100 *μ*l stop solution was added to stop the reaction. The ELISA plate was read by ELISA reader at 405 nm wavelength of filter absorbance (optical density OD/absorbance value) within 15 minutes after adding the stop solution.

### 2.6. Statistical Analysis

The relative level of antibody in the sample was determined by calculating the sample to positive (S/P) ratio. The endpoint titers were calculated using the equation described by the manufacturer. Serum samples with S/P ratio of less than or equal to 0.2 were considered negative, and those samples with S/P ratio greater than 0.20 (titer >396) were considered positive. The results of HI titers of all sera thus obtained were statistically analyzed using chi-square analysis at *p* < 0.05 level of significance. The chi-square analysis was used to compare the serotypes in each LGA to determine if there is a difference in serotypes across the LGAs. Risk factors potentially associated with IBV (i.e., age, breed of chicken, farm type, and flock size) were assessed by logistic regression and conducted using the SPSS version 20 software.

### 2.7. Ethics Approval

The animal handling and sample collection methods were performed in accordance with the Addis Ababa University College of Veterinary Medicine research ethics (AAU-CVMA-RE) and the animal welfare guide for the care and use of animals (Ref. No. VM/ERC/32/07/22/2021).

## 3. Results

### 3.1. Seroprevalence of Infectious Bronchitis Virus

A total of 354 serum samples were collected from six different farms (four smallholder and two commercial farms) found in central Ethiopia. All collected serum samples were undertaken for an indirect ELISA test and resulted an overall prevalence of 97.46%. The prevalence of IBV was highly significant (*p* ≤ 0.01; *χ*^2^ = 19.9) at the study site (Table 1).

From 230 and 124 samples collected from commercial and smallholder poultry farms, 226 (98.26%) and 119 (95.98%) positive results were obtained, respectively. Among the production of chickens, high seroprevalence (99.31%) was found in layer poultry, and lower seroprevalence (96.17%) was found in the case of broiler chicken (Table 2).

### 3.2. Analysis of Risk Factors Associated with Seroprevalence of IBV Infection

Prevalence of IBV infection was also assessed based on the different associated risk factors. Accordingly, the higher (100%) prevalence was observed in male than female chickens. Bovans Brown breeds of chicken were 100% positive to IBV infection in the study area. According to the type of farm analysis, there were higher seroprevalence of IBV infection in commercial type of production in the study area, but there was no significant association (*p* > 0.05; *χ*^2^ = 19.9) (Table 3).

## 4. Discussion

The cross-sectional study was carried out on serum samples of 354 chickens selected from six different farms to detect seroprevalence of IBV. No vaccines were given against IBV in all farms under the present study. The present study shows that overall prevalence of 97.46% infectious bronchitis virus was reported from selected flocks of six farms in central Ethiopia. This finding is in agreement with the report of Hutton et al. [17] who demonstrated overall prevalence (94.5%) of IBV in the poultry farm of Ethiopian Institute of Agricultural Research (EIAR). The report also agreed with the report of Ijoma et al. [5] surveyed on seroprevalence and serotypes of infectious bronchitis virus in free-range chickens in Plateau State, Nigeria, who reported an overall prevalence of 82.95%. Parallel survey was compared favorably to what was recorded in the southwestern part of Nigeria (82.7% [18], 84% [19]) and in the northern part of Nigeria, Sokoto (84%) [20]. This could suggest the possible carrier status of free-range chickens in the transmission of the virus.

This finding was also higher than the reports of Tesfaye et al. [14] and Yonas et al. [15] who reported 70.6% and 64.7% of seroprevalence of IBV in Sebeta Hawas and Ada'a districts, respectively. The difference in seroprevalence could be due to variations in agro-climatic conditions, sample size, and management systems. Moreover, the differences might be associated with an increase in the activity of IBV among chickens and birds in the study area [21].

However, the overall seroprevalence of IBV in this study is higher than the study conducted in Bangladesh (59.30%), Grenada (31.01%), and Maiduguri (26.6%) by Zafar et al. [7], Arathy et al. [22], and Shettima et al. [23], respectively. This may be due to the highly transmissible nature of the disease, its capacity to spread to a substantial distance through aerosol, and the presence of carriers in the environment [20]. In this study, the management problem and inconvenient data collection might be revealed the higher prevalence of the virus.

According to different sites from where the samples were collected, significantly highest seroprevalence of the IBV infection was observed in Holeta farms. These variations may be due to difference in sample size, management system, and burden of the virus in the study sites [5, 7, 8].

In the present study, age difference based on the status of the virus was observed, where it was found to be higher in adult chickens. This might be due to the difference in exposure toward the disease which is particularly true in the adult chickens [6, 24]. The range and magnitude of the serological results also provided evidence to suggest repeat exposure of birds to IBV supported by antigenic identification of both pathogens, suggesting circulation within the flocks. Again, increased biosecurity measures may be of assistance in reducing transmission but may be insufficient alone; due to the multiple ages of flocks, making complete disinfection of premises is impossible. Other management practices, such as ensuring adequate ventilation and reducing overcrowding, and the use of vaccination were necessary to reduce production losses [6].

In broiler and layer, the prevalence reported in this study is 95.69% and 100%, respectively. This report is in agreement with the statements of Jackwood [24] and Seger et al. [25] who reported higher prevalence of IBV in layer chickens. This different report tells us the difference in endemicity of the IBV in different study areas [6, 24].

Breed wise difference on the status of IBV infection was observed in the present study. Accordingly, significantly higher prevalence of the virus was observed in Bovans Brown breed. This might be due to the difference in seroprotection level of the disease or genetic difference of the two breeds against the disease [26].

## 5. Conclusion

IBD is complicating factor affecting poultry production systems in the area. Vaccine and biosecurity measures are recommended for the control of IBV. Furtherly, identification and characterization (by using RT-PCR) of persistent serotype of IBV circulating in the field are recommended. The authors also recommend further study by increased sample size, study periods, and study areas.

## Figures and Tables

**Table 1:** Prevalence of IBV per study sites.

Location	No. of sample collected	Positive	Prevalence (%)	*χ* ^2^	*p* value
Holeta	20	20	100		*p* ≤ 0.01
Bishoftu	334	325	97.30	19.9	
Total	354	345	97.46		

*χ* ^2^: chi-square.

**Table 2:** Total prevalence of IBV among farm and production types.

Factors	Level	No. of sample	No. of positive	Prevalence (%)	*χ* ^2^	*p* value
Farm type	Commercial	230	226	98.26%	1.7	0.28
	Small holder	124	119	95.98%		
Production type	Broiler	209	201.0	96.17%		
	Layer	145	144	99.31%		

*χ* ^2^: chi-square.

**Table 3:** Prevalence of IBV among different associated risk factors.

Factors		No. of sample	No. of positive	Seroprevalence	*χ* ^2^	*p* value
Farm type	Commercial	230	226	98.26%	1.70	0.28
	Small holder	124	119	95.98%		
Breed	Bovans Brown	145	145	100%	6.41	0.04
	Cobb 500	209	200	95.69%		
Age	Young	209	200	95.69%	6.41	0.04
	Adult	145	145	100%		
Sex	Male	64	64	100%	2.03	0.22
	Female	290	281	96.89%		
Production purpose	Broiler	209	200	95.69%	9.64	0.01
	Layer	145	145	100%		

*χ* ^2^: chi-square.

## Data Availability

The data used in this article is found with the corresponding author and required upon request.

## References

[1] Zeleke B. (2017). Status and growth trend of draught animals’ population in Ethiopia. *Journal of Dairy, Veterinary & Animal Research*.

[2] Lavinia P., Eduardo B. (2019). The importance of livestock and sustainable production systems. https://agrilinks.org/post/importance-livestock-and-sustainable-production-systems.

[3] Abdisa T., Tagesu T. (2017). Review on Newcastle disease of poultry and its public health importance. *Journal of Veterinary Science & Technology*.

[4] Mammo M. (2012). Chicken production scenarios and the headway options for improvement in Ethiopia. *World’s Poultry Science Journal*.

[5] Ijoma S., Shittu I., Chinyere C. (2021). Sero-prevalence and serotypes of infectious bronchitis virus in free-range chicken in Plateau state, Nigeria. *Sokoto Journal of Veterinary Sciences*.

[6] Cavanagh D., Gelb J., Saif Y. M., Fadly A. M., Glisson J. R., McDougald L. R., Nolan N. K., Swayne D. E. (2008). Infectious bronchitis. *Diseases of Poultry*.

[7] Giasuddin M. D., Bhuiyan Z. F., Mahmood Khan Z. U. (2018). Seroprevalence of infectious bronchitis virus in different types of chicken in Bangladesh. *Asian Journal of Medical and Biological Research*.

[8] Barua H., Bisuead P. K., Debnath N. C., Anwar M. N., Dey B. C. (2006). Serosurvey and isolation of infectious bronchitis virus in chicken reared in commercial and semi scavenging system. *Bangladesh Journal of Microbiology*.

[9] Cavanagh D. (2005). Coronaviruses in poultry and other birds. *Avian Pathology*.

[10] De-Wit J. J. (2014). *Detection of infectious bronchitis virus*.

[11] Priyanka M., Verma Y., Swamy M., Dubey A., Bharti A. (2019). Infectiou bronchitis in poultry: a review. *Journal of Entomology and Zoology Studies*.

[12] Sambo E., Bettridge J., Dessie T. (2014). Participatory evaluation of chicken health and production constraints in Ethiopia. *Preventive Veterinary Medicine*.

[13] Bellam P., Narasimha G. (2016). Detection of infectious bronchitis virus by enzyme linked immunosorbent assay (ELISA) in chickens. *Scholars Research Library Der Pharmacia Lettre*.

[14] Tesfaye A., Kassa T., Mesfin S. (2019). Four serotypes of infectious bronchitis virus are widespread in unvaccinated backyard chicken and commercial farms in Ethiopia. *World Journal of Veterinary Science*.

[15] Yonas T., Wabi B., Tsedale T., Asamnew T., Naol M., Fanos T. (2021). Serological detection of antibodies against gamma coronavirus infection in scavenging village chickens in Ada’a District, Ethiopia. *Biomedical Journal of Scientific & Technical Research*.

[16] Transitional government of ethiopia (2017). *volume I report on area and production of major crops private peasant holdings Meher season*.

[17] Hutton S., Bettridge J., Christley R., Habte T., Ganapathy K. (2017). Detection of infectious bronchitis virus, avian meta-pneumovirus. *Trop Anim Health Prod*.

[18] Emikpe B. O., Ohore O. G., Olujonwo M., Akpavie S. O. (2010). Prevalence of antibodies to infectious bronchitis virus (IBV) in chickens in southwestern Nigeria. *African Journal of Microbiology Research*.

[19] Owoade A. A., Ducatez M. F., Muller C. P. (2006). Seroprevalence of avian influenza virus, infectious bronchitis virus, reovirus, avian pneumovirus, infectious laryngotracheitis virus, and avian leukosis virus in Nigerian poultry. *Avian Diseases*.

[20] Mungadi H. U., Mera U. M., Adamu Y. A., Musa U., Achi C. R. (2015). Sero-prevalence of infectious bronchitis antibodies in local chickens in live bird markets in Sokoto State, Nigeria. *Scientific Journal of Animal Science*.

[21] Adene D. F. (2007). *The cornerstones in poultry health and production: concepts, costs and the contemporary applications*.

[22] Sabarinath A., Sabarinath G. P., Tiwari K. P., Kumthekar S. M., Thomas D., Sharma R. N. (2011). Seroprevalence of infectious bronchitis virus in birds of Grenada. *International Journal of Poultry Science*.

[23] Shettima Y. M., El-Yuguda A. D., Zanna M. Y. (2016). Serological evidence of infectious bronchitis virus among some poultry species in Maiduguri, Nigeria. *Alexandria Journal of Veterinary Sciences Alexandria*.

[24] Jackwood M. W. (2012). Review of infectious bronchitis virus around the world. *Avian Diseases*.

[25] Abdel-Moneim A. S., El-Kady M. F., Ladman B. S., Gelb J. (2006). S1 gene sequence analysis of a nephropathogenic strain of avian infectious bronchitis virus in Egypt. *Virology Journal*.

[26] Gharaibeh S., Mahmoud K. (2013). Decay of maternal antibodies in broiler chickens. *Poultry Science*.

